# Bilateral cavernous sinus and ophthalmic vein thrombosis secondary to *Trueperella pyogenes* rhinosinusitis

**DOI:** 10.1007/s12070-024-04505-1

**Published:** 2024-02-07

**Authors:** Lea Calo’, Emanuele Scarano, Francesca Brigato, Giovanni Di Cintio, Jacopo Galli

**Affiliations:** 1 Fondazione Policlinico Universitario Agostino Gemelli-Roma, Rome, Italy; 2 Head and Neck Department, IRCCS - Fondazione Policlinico Universitario Agostino Gemelli Roma, Rome, Italy; 3 Ospedale Cardinale G. Panico Tricase, Lecce, Italy; 4 Nuovo Ospedale Degli Infermi, Ponderano Biella, Italy

**Keywords:** Ophthalmic vein thrombosis, *Trueperella pyogenes*, Orbital cellulitis, Cavernous Sinus Thrombosis

## Abstract

Ophthalmic vein thrombosis is a severe clinical entity with proptosis, eyelid swelling, orbital pain and reduction of visual acuity; its incidence is rare with 3–4 cases /million /year. Clinical manifestations result from venous congestion caused by septic (orbital cellulitis) or aseptic aetiologies (coagulopathies, trauma) and in some cases it could be associated with cavernous sinus thrombosis. In this paper, we describe a case report unique in the literature, of bilateral cavernous sinus and ophthalmic veins thrombosis due to both septic and aseptic causes characterized by unilateral sphenoid sinusitis sustained by *Trueperella pyogenes* infection. *Trueperella pyogenes* is an opportunistic animal pathogen, and its infections occur in both domestic and wild animals worldwide but are rare in humans; this is the first instance of human infection in the head and neck with an unknown hypercoagulable state.

## Introduction

Superior Ophthalmic vein thrombosis (SOVT) is a rare and fearsome condition that presents signs of venous congestion with ptosis, conjunctival chemosis and ophthalmoplegia; it occurs secondary to sinonasal diseases and may be associated with orbital cellulitis and cavernous sinus thrombosis; in some cases, it is related to aseptic status with stasis of blood flow caused by neoplasm with invasion of cavernous sinus and orbit, trauma, haematological disease, or hypercoagulable state; usually, SOVT follows the infection diseases from sinuses, orbit, teeth and the most frequent responsible microorganisms are the same ones associated to rhinosinusitis infections, *staphylococcus aureus* and *streptococci* and recently *Covid 19 infection* [1].

The presence of veins thrombosis, orbital cellulitis and cavernous sinus thrombosis together elevates the risk of blindness and a life-threatening situation and makes the differential diagnosis particularly difficult. Early intervention and a multi-disciplinary approach are the key factors for the right and successful management.

In this paper, we describe a case report, unique in the literature, of bilateral cavernous sinus and ophthalmic vein thrombosis due to both septic and aseptic causes characterized by unilateral sphenoid sinusitis sustained by *Trueperella pyogenes* infection. *Trueperella pyogenes* is an opportunistic animal pathogen, and its infections occur in both domestic and wild animals worldwide but are rare in humans; this is the first instance of human infection in the sinonasal cavities with an unknown hypercoagulable state.

## Case Presentation

**Fig. 1:**
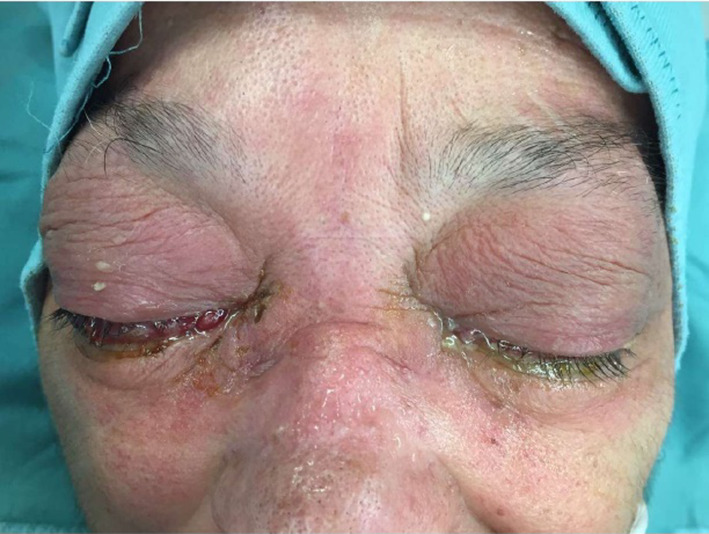
Eyelids oedema, bilateral ptosis, exophthalmos, purulent eye discharge

A 71-year-old woman a non-smoker, came to the Emergency Room with an high temperature (38 °C), complaining of headache and bilateral reduction of visual acuity. At the clinical examination, hyperaemia of the conjunctiva, eyelids oedema, bilateral ptosis, exophthalmos, and ophthalmoplegia were found, without other neurological signs (Fig. 1); rhinorrhoea and nasal obstruction were not observed. Covid test was negative, and she referred complete vaccine status, no comorbidity nor previous surgery. She did not report allergy to any medication, food or inhalant allergens; she had no previous history of rhinosinusitis, nasal polyps or ENT surgery.

**Fig. 2:**
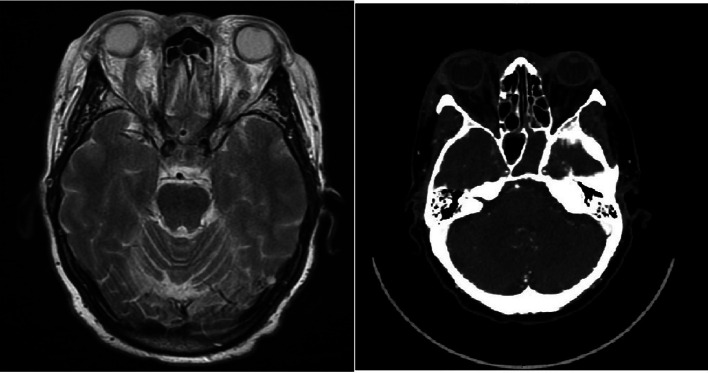
Imaging MRI and CT pre-treatment

Laboratory tests characterized by neutrophilic leucocytosis (16.16 × 109/L). Computed Tomography (CT) showed: “*complete thrombosis of the upper ophthalmic vein bilaterally, of the distal vein of the lower ophthalmic vein bilaterally and of both cavernous sinuses, which are not opacified either in the arterial or venous phase. It also documents partial thrombosis of the right vertebral vein and the jugular veins within the extracranial tract. Filiform internal carotid arteries in the intracavernous tract, however pervious, probably by ab-extrinsic compression from thrombosis of the cavernous sinuses*”; these findings were coherent for the diagnosis of sphenoid rhinosinusitis. These data were confirmed by Magnetic Resonance Imaging (MRI): “*complete thrombosis of ophthalmic veins and cavernous sinuses. The left sphenoid is engaged by tissue without enhancement but with restriction of proton diffusion, compatible therefore also with high cellularity tissue. The Sphenoid is completely engaged but not deformed. It is not possible to exclude a mycotic localization*” (Fig. 2). The patient was treated with anticoagulant therapy, i.e. enoxaparin sodium (6000 IU bid), and intravenous antimicrobial therapy, i.e. vancomycin (1gr bid), ceftazidime (2gr tid), metronidazole (500 mg tid), and fluconazole (400 mg/die). Because of the unsatisfactory clinical improvement obtained in the next 48 h with medical therapy, the patient underwent surgical treatment. An endoscopic sinus surgery was performed and a left sphenoidotomy was carried out. The pus and the pathological mucosa were examined: microbiological assessment revealed colonization by *T. pyogenes* without mycotic components.

Given the microbiological results, investigating the causes of colonization by *T. pyogenes*, we discovered that the patient lived in a rural environment with 40 dogs and 27 cats. During post-operative hospitalization, therapy with vancomycin, ceftazidime, metronidazole and fluconazole was interrupted and substituted by ceftriaxone (2 gr/die), according to the antibiogram and the minimal inhibitory concentration (MIC) evaluation. The patient presented a progressive improvement of signs and symptoms until complete resolution; and no more venous congestion at fundus oculi examination was observed.

**Fig. 3:**
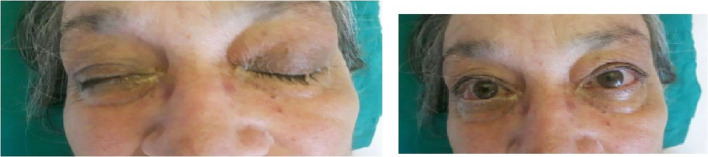
Complete resolution of the eyelids  oedema, ptosis  and purulent discharge post-surgical and antibiotic treatment

At follow-up at twelve months the patient is healthy, but extensive hematologic tests showed an unknown hypercoagulable state, factor V Leiden mutation, so she is still in antithrombotic therapy (Fig. 3).

## Discussion

*Trueperella pyogenes*, genera Arcanobacterium, is a gram-positive, non-motile, non-sporulating and polymorphic bacterium and it is a worldwide known opportunistic pathogen of domestic animals associated with suppurative or granulomatous lesions. Clinical animal manifestations are various, with the predominance of mastitis (45.1%), abscesses (18%), pneumonia (11.1%), and lymphadenitis (9%). Infections of human patients with *T. pyogenes* are rare, mostly related to living in rural areas and contact with animals [1–4]. Not more reports of *T. pyogenes* infections in humans are described since its discovery over a century ago, and specifically only a single case in head and neck: otitis with consequent brain abscess [5].

*Trueperella pyogenes* is often misidentified and underdiagnosed, only in 2011 it was named *Trueperella,* differentiated from the other common pyogenes agents: *Corinaebacteria, Actinomyces* and *Arcanobacteria*, thanks to revision based on new chemotaxonomic data.

Until now, it has not been isolated in healthy human microbiomes. Virulence mechanisms related to *Trueperella* pyogenes are due to cytolytic activities associated with transmembrane pore formation and immune cells.

Pyolysin (PLO) is a major known virulence factor *of T. pyogenes* that belongs to the family of cholesteroldependent cytolysins able to form pores in the cholesterol-containing membrane and consequent loss of intracellular K + . The loss of intracellular K + can lead to the expression of the proinflammatory cytokine, IL-1β, playing an essential role in the occurrence of *T. pyogenes-*related inflammation [6]. Other putative virulence factors, including neuraminidases, extracellular matrix-binding proteins, fimbriae, and biofilm formation ability, contribute to the adhesion and colonization of the host tissues [6–8]. The isolation of *T. pyogenes* in the sinonasal cavities is unknown; this is the first case report with complicated bilateral ophthalmic veins and cavernous sinus thrombosis secondary to *T. pyogenes* infection, and the site of infection was identified in restricted unilateral sphenoid sinusitis.

The human contamination probably occurred through contact with animals’ skin, perhaps in our case *T. pyogenes* virulence was related to the adhesion and colonization of the host tissues resulting from its extracellular matrix-binding proteins, fimbriae, and biofilm formation ability after upper airways pollution. The microorganism’s biofilm formation ability plays an important role in rhinosinusitis. The diffusion into the sphenoid and the consequent cytolysis activities against the cellular membranes completed the infection pyogenic process. The infection then spread through the venous sinuses and the orbit in a patient affected by thrombophilia predisposition, which was unknown until then.

Moreover, the association between thrombotic phenomena and *T. pyogenes* infection is described in both animals and humans. At present, the pathogenic mechanisms that underlie the onset of thrombotic phenomena remain unknown, even if some antigens may play a key role in the activation of coagulation factors. Deliwala et al. described a case of infective endocarditis by *T. pyogenes* complicated by an ischemic spinal vasculopathy, emphasizing the systemic implications underneath. In fact, septic thromboembolism involving the brain, kidneys, lungs, and endocarditis were documented [6]. In consideration of these case reports, the echocardiographic evaluation became noticeable in our patient to exclude the cardiogenic origin of possible septic thromboembolism; laboratory tests and imaging confirmed the diagnosis of ophthalmic venous thrombosis as a single clinical sign of unilateral sphenoid sinusitis.

*T. pyogenes* could be also associated with wound infections; one paper described three cases that occurred in India emphasizing the bacteriological characteristics which were relevant for the identification of these bacteria and that all the patients lived in a rural area in close contact with the cattle.

*T. pyogenes* has been reported as the causative organism for human disease including septic arthritis, empyema, vulvovaginitis; moreover one case of acute meningitis with isolation of this micro-organism from cerebrospinal fluid was described in a 76-year-old man who had worked with livestock [5].

Unfortunately, the clinical presentation can be variable, and infection signs appear non-specific with respect to other bacteria.

*T. pyogenes* was never identified as a commensal microbiota of humans, those infections should be considered as zoonotic diseases, and the rare cases reported may be proof that animal-to-human transmission of this pathogen has not been well estimated and confirmed.

It is important to remember that zoonosis has no common signs and symptoms involving the head and neck, and ENT specialists should consider the possibility of a zoonotic disease in differential diagnosis, particularly in cases not responding to common therapy. It should be important to pay attention to a proper anamnesis, for example investigating conditions of immunosuppression, professional exposure (farmer, veterinarian, hunter), the environment of life, recent travels in endemic regions, and eating habits. Diagnostic doubt must also guide the choice of the most appropriate examinations to identify the cause of the disease.

## Take Home Messages

- *T. pyogenes* is an established, but often misrecognized pathogen that should be better considered by clinical microbiologists. Even if, most aspects of the pathogenic activity are already obscure and the scientific data are limited concerning the effects on coagulation, *T. pyogenes* could have a direct role in the induction of thrombotic activity, despite a non-diffusive infectious process.
- It is important to remember that zoonosis has no common signs and symptoms involving the head and neck; then ENT specialists should consider the possibility of a zoonotic disease to achieve a correct diagnosis, particularly in cases not responding to common therapy.
- Animal-to-human and human-to-human transmission has never been estimated, although the possibility of an endemic spread is possible, as the *Corynebacterium pyogenes* in epidemic leg ulcers in Thailand in 1979 and 1980.
- The clinical course of these suppurative infections may be severe, with high mortality rates in case of misdiagnosis and inappropriate treatment
